# A taste for ATP: neurotransmission in taste buds

**DOI:** 10.3389/fncel.2013.00264

**Published:** 2013-12-18

**Authors:** Sue C. Kinnamon, Thomas E. Finger

**Affiliations:** ^1^Department of Otolaryngology, Rocky Mountain Taste and Smell Center, University of Colorado School of MedicineAurora, CO, USA; ^2^Department Cell and Developmental Biology, Rocky Mountain Taste and Smell Center, University of Colorado School of MedicineAurora, CO, USA

**Keywords:** purinergic, P2Y, adenosine, glossopharyngeal, chorda tympani, hemichannel, pannexin, connexin, CALHM1

## Abstract

Not only is ATP a ubiquitous source of energy but it is also used widely as an intercellular signal. For example, keratinocytes release ATP in response to numerous external stimuli including pressure, heat, and chemical insult. The released ATP activates purinergic receptors on nerve fibers to generate nociceptive signals. The importance of an ATP signal in epithelial-to-neuronal signaling is nowhere more evident than in the taste system. The receptor cells of taste buds release ATP in response to appropriate stimulation by tastants and the released ATP then activates P2X2 and P2X3 receptors on the taste nerves. Genetic ablation of the relevant P2X receptors leaves an animal without the ability to taste any primary taste quality. Of interest is that release of ATP by taste receptor cells occurs in a non-vesicular fashion, apparently via gated membrane channels. Further, in keeping with the crucial role of ATP as a neurotransmitter in this system, a subset of taste cells expresses a specific ectoATPase, NTPDase2, necessary to clear extracellular ATP which otherwise will desensitize the P2X receptors on the taste nerves. The unique utilization of ATP as a key neurotransmitter in the taste system may reflect the epithelial rather than neuronal origins of the receptor cells.

## INTRODUCTION

Epithelia are faced with seemingly conflicting tasks – first, they serve as a barrier between the external world and the innards of an organism, and second, they are extended sensory organs responding to varied stimuli in the external world including temperature, pressure, and even illumination. The epithelial barrier is formed by junctional complexes between epithelial cells obstructing the free diffusion of materials from outside to in. The sensory functions are accomplished either by direct responses by keratinocytes, or by activation of appropriate sensors on intraepithelial nerve fibers. The sensory responses by keratinocytes are relayed to the sensory nerve fibers by release of appropriate mediators or transmitters including ATP, which activates neural purinergic receptors (e.g., Mandadi et al., 2009; Barr et al., 2013). Thus purinergic signaling is a common means by which epithelial keratinocytes communicate with sensory nerve fibers. In contrast, typical epithelial sensory endorgans, e.g., photoreceptors, auditory hair cells, and olfactory receptor cells, utilize conventional neurotransmitters such as glutamate, for neurotransmission.

Taste buds, the sensory endorgans of gustation consist of a collection of 50–100 specialized, columnar taste cells embedded in the relatively non-specialized, lightly keratinized stratified squamous lingual epithelium. The keratinocytes of the non-specialized lingual epithelium are similar to epithelial cells elsewhere in the body in that they respond to a variety of external stimuli, e.g., pressure, or chemicals, by releasing ATP along with other intercellular signaling molecules (Mandadi et al., 2009; Lazarowski et al., 2011; Barr et al., 2013). Taste cells, like other epithelial cells, but unlike other epithelial sensory endorgans, rely on ATP to activate the sensory nerves innervating the taste buds (Bo et al., 1999; Finger et al., 2005). However, although taste cells have an epithelial origin, they do have neuron-like voltage-gated ion channels and generate action potentials to most taste stimuli (Damak et al., 2006; Yoshida et al., 2009). While depolarization is required for ATP release and the amount of ATP released is proportional to the frequency of action potentials (Murata et al., 2010), the requirement for action potentials in ATP release is controversial (Huang and Roper, 2010).

ATP is utilized for intercellular communication in a wide variety of biological contexts including neural signaling. At many synapses, ATP is co-released with a conventional neurotransmitter and serves in a trophic or modulatory role modifying the responsivity of the sensory cells or modifying actions of a conventional neurotransmitter. For example, in the auditory system, ATP serves a protective function helping maintain epithelial integrity in the face of extreme stimulation (Thorne et al., 2004). In the olfactory system, ATP modulates neural sensitivity, induces production and release of growth factors, and modulates cell division of proliferative basal cells (Jia et al., 2011). In these systems, interruption of purinergic signaling leads to relatively minor disruption of function or cell turnover. Conversely, in the taste system, ATP is necessary for transmission of information from the sensory cells to the afferent nerve fibers. Genetic elimination of the P2X receptors on the sensory nerve fibers (P2X2 and P2X3) totally eliminates transmission of the signal from taste receptor cells to nerve fibers (Finger et al., 2005). In this review, we describe the evidence that ATP serves a unique, crucial role in transmission of taste information from the taste buds to the taste nerves.

## TASTE BUDS

Taste cells, the sensory cells of taste buds, arise during late embryogenesis, not from neural progenitors such as neural crest or neurogenic placodes, but from the lingual epithelium itself (Stone and Finger, 1994; Barlow and Northcutt, 1995). Thus, unlike other receptor cells, e.g., olfactory receptor cells and hair cells, taste cells are a specialized component of the epithelium rather than being derived from neurogenic progenitors. Molecular differentiation of the undifferentiated lingual epithelium begins about day E12 in mice, and can first be recognized by small clusters of cells expressing a variety of developmental signaling molecules such as sonic hedgehog, wnts, and BMP (Hall et al., 1999; Jung et al., 1999; Mistretta et al., 2003; Iwatsuki et al., 2007; Liu et al., 2007; Kapsimali and Barlow, 2013). Fully differentiated taste cells first appear shortly before birth although full elaboration of the peripheral taste system does not occur until several days postnatally.

**FIGURE 1:**
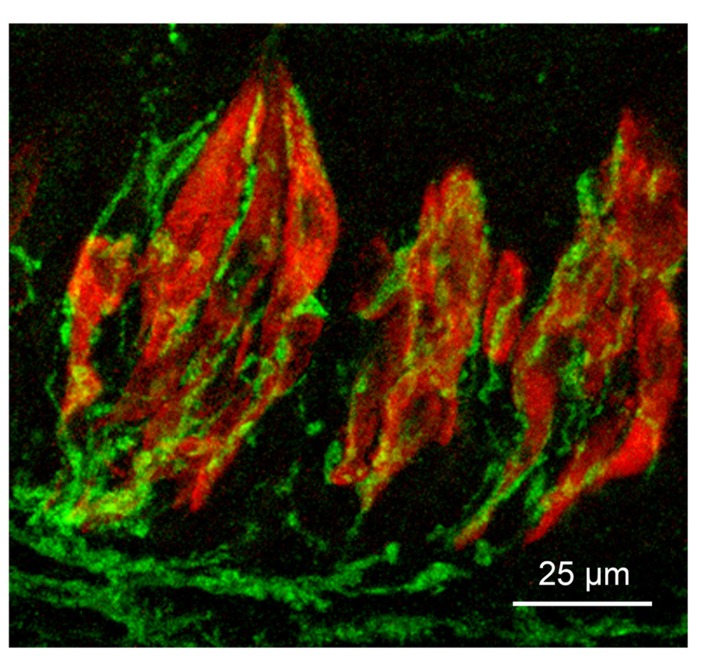
**Micrograph of longitudinal section through three taste buds in the circumvallate papilla of a mouse**. Type II taste cells are stained red with antiserum to PLCβ2; gustatory afferent fibers are stained green with an antiserum to P2X3. The surface of the epithelium is at the top of the micrograph.

A mature taste bud contains multiple types of taste cells distinguished morphologically, physiologically, and molecularly (**Figure 1**). Conventionally, taste cells are divided into three types, which although based originally on staining characteristics, correlates well with molecular and functional expression profiles (Yee et al., 2001; Finger, 2005; Finger et al., 2005; Huang et al., 2007; Romanov et al., 2007; Roper, 2013). *Type I cells*, which constitute the majority of cells within each bud, are glial-like in that they exhibit several features common to astrocytes. They enwrap other cells with flattened processes, express proteins associated with neurotransmitter reuptake or catabolism (including NTPDase2; Bartel et al., 2006), and form no apparent specialized contacts with the sensory nerve fibers. *Type II cells* are the receptor cells for the taste qualities of sweet, bitter, and umami (savory) mediated by the taste receptor (TR) family of G-protein coupled taste receptors and the related phospholipase C (PLC)-mediated downstream cascade (Yarmolinsky et al., 2009; Roper, 2013). The points of contact between the type II cells and nerve fibers often exhibit a non-conventional specialization involving subsurface cisternae and atypical mitochondria (Royer and Kinnamon, 1988; Clapp et al., 2004). No conventional synapses, complete with presynaptic vesicles and postsynaptic membrane thickening are evident at contacts between type II cells and nerve fibers. *Type III cells* do not express the TR family taste receptor proteins or downstream cascade, but do form conventional synapses with the afferent nerves (Royer and Kinnamon, 1988). Type III cells are required for sour taste transduction but are not required for transmission of taste information from type II cells to the afferent nerves since genetic deletion of these cells does not disrupt sweet, bitter, and umami detection (Huang et al., 2006).

The cellular basis of salt taste is poorly understood but likely involves multiple cell types and mechanisms (Chandrashekar et al., 2010; Oka et al., 2013). High concentrations of salt stimulate both type III cells and type II cells via an amiloride-insensitive mechanism (Oka et al., 2013). In contrast, low concentrations of salt primarily utilize the amiloride-sensitive epithelial sodium channel, ENaC, in a cell type (Chandrashekar et al., 2010), lacking voltage-gated ion channels (Vandenbeuch et al., 2008), i.e., not a typical type II or type III cell. If these ENaC-expressing taste cells are type I cells, as suggested by Vandenbeuch et al. (2008), how these glial-like taste cells might communicate with the afferent nerve fibers is unclear. One possibility is that ENaC-expressing taste cells communicate via a paracellular mechanism to electrically excitable cells in the taste bud which relay the signal to the nerve fibers.

Since type III cells are the only cells that possess conventional synapses, then transmission of taste information from type II cells to nerve fibers must utilize a non-conventional functional contact, perhaps the contacts with subsurface cisternae and specialized mitochondria.

## NEUROTRANSMITTERS IN TASTE CELLS

Several potential neurotransmitters have been identified in taste buds (for detailed review, Roper, 2013). These include serotonin (Kaya et al., 2004; Huang et al., 2005, 2009), GABA (Cao et al., 2009; Starostik et al., 2010; Dvoryanchikov et al., 2011; Huang et al., 2011a), and noradrenalin (Huang et al., 2008; Zhang et al., 2010), which are released from type III cells, and acetylcholine, released from type II cells (Dando and Roper, 2012). In addition, several peptide transmitters have been identified in taste buds (for recent review, Dotson et al., 2013). These include CCK, VIP, NPY, and PYY, and glucagon in type II cells, and GLP-1 and galanin in both type II and type III cells. Ghrelin appears to be expressed non-specifically in all taste cells. Rather than primarily activating afferent nerve fibers, these transmitters and peptides appear to exert their effects largely by binding to cognate receptors on adjacent taste cells, modulating the output of the taste bud. The possible exceptions to this are serotonin, which may activate 5-HT3 receptors on afferent nerve fibers (Kaya et al., 2004) and GLP-1, which may activate GLP-1 receptors on both nerve fibers and other taste cells to modulate sweet taste (Shin et al., 2008). However, knockout (KO) of either 5-HT3 (Finger et al., 2005) or GLP-1R (Shin et al., 2008) fails to block taste behaviors, suggesting that while these transmitters may play a role in activating nerve fibers, they are not required.

Glutamate also has been suggested to serve as a taste transmitter (Vandenbeuch et al., 2010a), primarily because the glutamate transporter GLAST is co-expressed in type I taste cells (Lawton et al., 2000). However, the expression of vesicular glutamate transporters VGLUT1 and 2 is restricted to afferent nerve fibers (Vandenbeuch et al., 2010a), suggesting glutamate may be released from afferent nerve fibers via an axon reflex, thereby modulating taste bud function by activating ionotropic glutamate receptors on the type III taste cells (Caicedo et al., 2000; Vandenbeuch et al., 2010a; Niki et al., 2011; Huang et al., 2012). Although all of these potential transmitters may play roles in modulating taste, none has been shown to meet *all* of the criteria for a substance to be accepted as an afferent transmitter: presence in the presynaptic cell, release upon stimulation, activation of postsynaptic receptors on afferent nerve fibers, and a mechanism for degradation or removal of the transmitter from the extracellular space. Only ATP meets all four of these criteria.

## ATP RELEASE

ATP is present at mM concentrations in the cytoplasm of all cells, so the question is, whether ATP is released by taste stimulation, and if so, by what mechanisms? ATP release with taste stimulation was first described at a tissue level by a luciferin–luciferase assay (Finger et al., 2005) and subsequently characterized at a cellular level by a variety of techniques, including biosensor cells containing purinergic receptors (Huang et al., 2007; Romanov et al., 2007), and luciferin–luciferase assays from patch pipets contacting identified taste cells (Murata et al., 2010). All of these studies suggested that release occurred by an unconventional, non-vesicular mechanism likely involving depolarization-activated ATP release channels. Curiously, release was only detected from type II taste cells, i.e., those that lack conventional synapses with the afferent nerve fibers. The identity of the ATP release channel is still in question, since several putative release channels are expressed in taste buds: connexins 43 and 30 in both taste cells and non-gustatory epithelial cells (Huang et al., 2007; Romanov et al., 2007), pannexin-1, expressed primarily in type II cells (Huang et al., 2007; Romanov et al., 2007), and a recently discovered ATP release channel, CALHM1, expressed in most type II taste cells (Taruno et al., 2013). The only channel knockout that has been examined at the systems level in taste buds is CALHM1, which shows severely diminished responses to bitter, sweet, and umami taste stimuli (all type II cell qualities), with little effect on other qualities. These data suggest that CALHM1 plays a role in the release process. However, the pharmacology of taste-evoked release suggests the ATP release channels are likely composed of pannexin-1, since release is blocked by low concentrations of carbenoxolone (Huang et al., 2007, 2011b; Dando and Roper, 2009; Murata et al., 2010). Taste buds of pannexin-1 knockouts are capable of ATP release (Romanov et al., 2012), but these knockouts have not been examined with either taste nerve recording or behavior, so the mechanism of release remains in question.

## PURINERGIC RECEPTORS IN AFFERENT FIBERS AND TASTE BUDS

The presence of purinergic receptors on the afferent nerve fibers was first discovered by Bo et al. (1999), who found both P2X2 and P2X3 on nerve fibers innervating taste buds. This has been examined more recently by Ishida et al. (2009), who showed that all geniculate ganglion neurons in rodents express P2X3, with approximately 70% also expressing P2X2. If P2X2 and P2X3 are required for transmitting taste information to the nervous system, then the double knockout of P2X2 and P2X3 should abolish taste-evoked behavior. Indeed, not only were responses to sweet, bitter, and umami abolished in the double knockout, but responses to other taste stimuli were abolished as well, suggesting ATP was required for transmission of all taste qualities to the nervous system (Finger et al., 2005; Ohkuri et al., 2012). Interestingly, single knockouts of either P2X2 or P2X3 had only a minor taste phenotype, suggesting that either P2X2 or P2X3 is capable of forming functional homomeric receptors in the taste afferents (Finger et al., 2005) although the typical receptor in wildtype mice is likely a P2X2/P2X3 heteromer.

As is true for any genetic deletions, global knockout of P2X2 and P2X3 may affect development or carry other unintended effects that could negatively impact taste functions. Indeed, the P2X2/P2X3 double knockout mice fail to release ATP normally in response to taste stimulation, suggesting the lack of taste responses could be due to a presynaptic rather than a postsynaptic defect (Huang et al., 2011b). Nevertheless, our recent discovery of a pharmacological recapitulation of the knockout findings is further confirmation of the importance of purinergic signaling in taste transmission. After i.p. injection of an antagonist selective for P2X3-containing receptors (Vandenbeuch et al., 2013a), responses to all taste qualities are eliminated or substantially reduced. The lack of responses to sour and salty stimuli as in the double P2X knockout is especially noteworthy since ATP release has not been detected from type III cells in response to either depolarization or sour stimuli (Huang et al., 2007; Romanov et al., 2007). However, recent studies indicate that nerve fibers contacting type III cells do express P2X2 (Yang et al., 2012) and presumably P2X3 as well (based on Ishida et al., 2009), so the morphological substrate for ATP signaling from type III cells is present.

**FIGURE 2:**
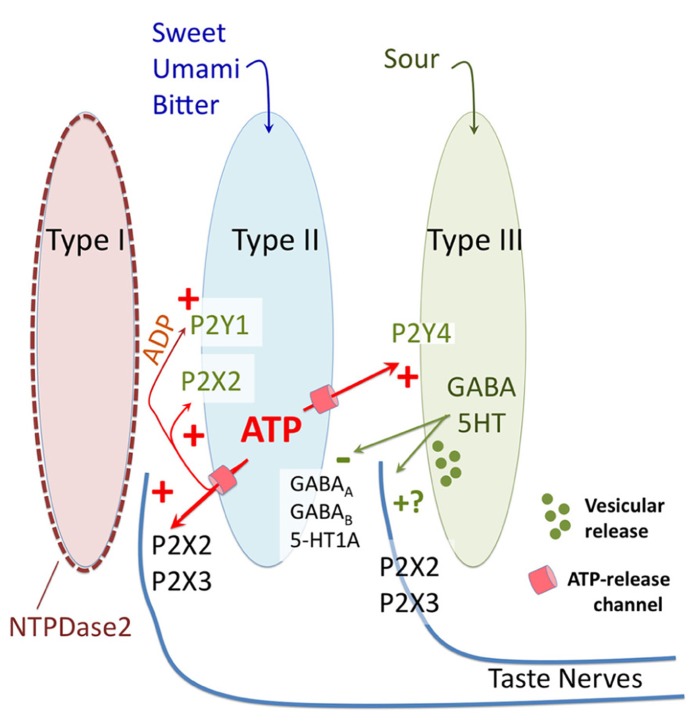
**Diagrammatic representation of purinergic signaling in taste buds**. In response to bitter, sweet, and umami stimuli type II taste cells release ATP via non-vesicular release channels. The released ATP activates afferent nerve fibers by binding to ionotropic receptors containing P2X2 and P2X3 subunits. The released ATP is hydrolyzed to ADP by a specific ectoATPase, NTPDase2, expressed on the membranes of type I taste cells. In addition to activation of afferent nerves, the released ATP (and its breakdown product ADP) activate purinergic receptors (P2X2 and P2Y1) on the type II cells themselves to potentiate further ATP release. ATP also activates the sour-sensitive type III taste cells via P2Y4, causing vesicular release of 5HT and possibly GABA, which in turn inhibits further ATP release from the type II taste cells via a paracrine feedback mechanism. Unclear is whether the 5HT and GABA also activate the afferent nerve fibers, and what the source of ATP is for sour taste, since all taste qualities require ATP for activation of afferent fibers.

P2X receptors also are present on taste cells themselves (**Figure 2**). P2X2 is present on the membranes of type II taste cells (Hayato et al., 2007; Huang et al., 2011b), where it offers a positive feedback loop for potentiation of ATP release. Other P2X receptors identified in taste tissue by RT-PCR include P2X4 and P2X7 (Hayato et al., 2007), although the functional significance of these receptors is not clear. Taste cells also possess metabotropic P2Y receptors, as first documented by calcium imaging studies showing ATP-induced calcium responses in taste cells that are mediated by release of calcium from intracellular stores (Baryshnikov et al., 2003). Several isoforms have been identified by molecular and pharmacological approaches, including P2Y1, P2Y2, and P2Y4 (Kataoka et al., 2004; Bystrova et al., 2006; Huang et al., 2009). P2Y1 is expressed primarily on type II cells, where it potentiates the release of ATP, while P2Y4 is expressed on type III cells, where it stimulates the release of 5-HT in response to ATP release from type II cells (Huang et al., 2009).

## CLEARANCE OF ATP RELEASED FROM TASTE BUDS

All neurotransmitter systems have mechanisms for either uptake or degradation of transmitter following release. In taste buds this is accomplished by a single ectoATPase, NTPDase2, expressed on the membranes of type I taste cells (Bartel et al., 2006). When ATP is released from taste cells, NTPDase2 degrades the ATP to ADP, which is further degraded to adenosine by other less specific ectonucleotidases including ecto-5’-nucleotidase expressed in type III cells (Dando et al., 2012). Mice globally lacking NTPDase2 have highly elevated levels of ATP in the extracellular space surrounding taste buds (Vandenbeuch et al., 2013b). The increased levels of ATP cause severely diminished taste responses to most taste stimuli, including sour stimuli that activate type III taste cells. The diminished responses are likely caused by desensitization of rapidly adapting P2X3-containing receptors on the afferent fibers. As described above, the co-expression of P2X2 and P2X3 in the majority of taste ganglion cells implies that most P2X receptors on the nerve fibers will be P2X2/P2X3 heteromers. However, we cannot rule out other possibilities for reduced taste function in NTPDase KO animals, such as inhibition of the ATP release channel by high levels of extracellular ATP (Qiu and Dahl, 2009). Nonetheless, these NTPDase2 knockout data provide further support for the requirement of ATP for all taste qualities, although the cellular source of the ATP for sour and salty stimuli remains enigmatic.

The adenosine that is ultimately produced by the degradation of ATP also modulates taste function. Of the known receptors for adenosine, only the A2B receptor is expressed in taste buds. The receptor is specifically expressed in posterior tongue, on the subset of type II taste cells that expresses sweet taste receptors (Dando et al., 2012; Kataoka et al., 2012). Adenosine enhances ATP release in response to sweet stimuli, thereby potentiating sweet responses (Dando et al., 2012). Knockout of the A2B receptor specifically diminishes sweet taste responses in the glossopharyngeal nerve, with no effect on other taste qualities (Kataoka et al., 2012).

## FUTURE DIRECTIONS AND CONCLUSIONS

Taste buds are unusual sensory endorgans in that they utilize ATP as the primary neurotransmitter connecting the sensory cells to the afferent nerve supply. In other special sensory systems ATP may be co-released with a conventional neurotransmitter, but it is not the primary substance necessary for neural communication (Housley et al., 2009). In contrast, in the carotid body, an interoceptive chemosensor, ATP does play a crucial role in transmission of information from the chemoreceptor cells to the vagal afferent nerve terminals (Piskuric and Nurse, 2013). In this respect, taste buds are more similar to a visceral interoceptor than to special sensory modalities.

The carotid body and taste buds are similar in other ways as well. In both carotid body and taste buds, ATP release is effected at least in part via gated ion channels. Once released, the ATP gates P2X2 and P2X3 receptors on the afferent nerve fibers. Both carotid body and taste buds possess multiple cell types including a glial-like cell (type I cells in taste buds; type II cells in carotid body). Both endorgans also possess cells with diverse neurochemical characteristics in addition to the purinergic phenotype. But significant differences exist between these two systems. In carotid body, the glial-like cells have P2Y receptors, which amplify the initial purinergic signal by triggering additional release of ATP (Piskuric and Nurse, 2013). In taste buds, the glial-like cells express an ectoATPase whose function is to rapidly break down ATP in extracellular space.

Many features and functions of taste buds remain unexplained. Foremost is the fashion in which signal specificity is maintained. Each taste bud contains taste cells responding to each of the various taste qualities, i.e., different cells responsive to bitter, sweet, umami, sour, and salty (Tomchik et al., 2007; Yoshida et al., 2009). If all the cell types utilize the same neurotransmitter, i.e., ATP, how can the system maintain specificity? How can ATP released into the tight confines of a taste bud activate only a single class of nerve fiber? Do other neurotransmitters and neuropeptides expressed by the different types of taste cells contribute to specificity by activating only appropriately matched nerve fibers? For example do sweet-responsive taste cells release both ATP and GLP-1 so that only sweet-best taste nerve fibers, which express both P2X receptors and GLP-1 receptors (Shin et al., 2008), become activated maximally?

A second major unanswered question relating to purinergic signaling in taste buds, is how and if ATP is released by sour and salt-responsive type III taste cells which possess conventional synapses. Type III cells do release serotonin and GABA using a vesicular release mechanism (Vandenbeuch et al., 2010b; Huang et al., 2011a), but do they co-release ATP? Blockade or abolition of P2X receptors prevents transmission of sour taste information, yet no one has yet succeeded in measuring ATP release from type III taste cells. Might type III taste cells excite type II cells to release ATP via hemichannels, similar to the way that type I carotid body cells excite type II carotid cells to release ATP via hemichannels? Alternatively ATP might be co-released via a vesicular mechanism along with serotonin or GABA, but the type III taste cells which possess classical synapses do not express vesicular nucleotide transporter (VNUT), the vesicular transporter for ATP (Iwatsuki et al., 2009). Hence it is unclear how the ATP would be packaged into the synaptic vesicles in this system.

Although taste buds contain fewer than 100 cells, they remain a complex and enigmatic endorgan. Clearly ATP and P2X receptors play a crucial role in linking taste buds to the afferent nerves. The recent decades of intense anatomical, physiological, and molecular characterization have permitted elucidation of many of the fundamental principles of taste bud organization, but much remains to be explained. These seemingly simple, small sensory endorgans remain a rich field for future studies.

## Conflict of Interest Statement

The authors declare that the research was conducted in the absence of any commercial or financial relationships that could be construed as a potential conflict of interest.

## References

[B1] Barlow L. A. Northcutt R. G. (1995). Embryonic origin of amphibian taste buds. *Dev. Biol.* 169 273–285 10.1006/dbio.1995.1143 7750643

[B2] Barr T. P. Albrecht P. J. Hou Q. Mongin A. A. Strichartz G. R. Rice F. L. (2013). Air-stimulated ATP release from keratinocytes occurs through connexin hemichannels. *PLoS ONE* 8:e56744 10.1371/journal.pone.0056744 PMC3574084 23457608

[B3] Bartel D. L. Sullivan S. L. Lavoie E. G. Sevigny J. Finger T. E. (2006). Nucleoside triphosphate diphosphohydrolase-2 is the ecto-ATPase of type I cells in taste buds. *J. Comp. Neurol.* 497 1–12 10.1002/cne.20954 16680780 PMC2212711

[B4] Baryshnikov S. G. Rogachevskaja O. A. Kolesnikov S. S. (2003). Calcium signaling mediated by P2Y receptors in mouse taste cells. *J. Neurophysiol.* 90 3283–3294 10.1152/jn.00312.2003 12878712

[B5] Bo X. Alavi A. Xiang Z. Oglesby I. Ford A. Burnstock G. (1999). Localization of ATP-gated P2X2 and P2X3 receptor immunoreactive nerves in rat taste buds. *Neuroreport* 10 1107–1111 10.1097/00001756-199904060-00037 10321492

[B6] Bystrova M. F. Yatzenko Y. E. Fedorov I. V. Rogachevskaja O. A. Kolesnikov S. S. (2006). P2Y isoforms operative in mouse taste cells. *Cell Tissue Res.* 323 377–382 10.1007/s00441-005-0098-8 16328495

[B7] Caicedo A. Jafri M. S. Roper S. D. (2000). In situ Ca^2^^+^ imaging reveals neurotransmitter receptors for glutamate in taste receptor cells. *J. Neurosci.* 20 7978–7985 11050118 10.1523/JNEUROSCI.20-21-07978.2000 PMC6772752

[B8] Cao Y. Zhao F. L. Kolli T. Hivley R. Herness S. (2009). GABA expression in the mammalian taste bud functions as a route of inhibitory cell-to-cell communication. *Proc. Natl. Acad. Sci. U.S.A.* 106 4006–4011 10.1073/pnas.0808672106 19223578 PMC2656195

[B9] Chandrashekar J. Kuhn C. Oka Y. Yarmolinsky D. A. Hummler E. Ryba N. J. (2010). The cells and peripheral representation of sodium taste in mice. *Nature* 464 297–301 10.1038/nature08783 20107438 PMC2849629

[B10] Clapp T. R. Yang R. Stoick C. L. Kinnamon S. C. Kinnamon J. C. (2004). Morphologic characterization of rat taste receptor cells that express components of the phospholipase C signaling pathway. *J. Comp. Neurol.* 468 311–321 10.1002/cne.10963 14681927

[B11] Damak S. Rong M. Yasumatsu K. Kokrashvili Z. Perez C. A. Shigemura N. (2006). Trpm5 null mice respond to bitter, sweet, and umami compounds. *Chem. Senses* 31 253–264 10.1093/chemse/bjj027 16436689

[B12] Dando R. Dvoryanchikov G. Pereira E. Chaudhari N. Roper S. D. (2012). Adenosine enhances sweet taste through A2B receptors in the taste bud. *J. Neurosci.* 32 322–330 10.1523/JNEUROSCI.4070-11.2012 22219293 PMC3566648

[B13] Dando R. Roper S. D. (2009). Cell-to-cell communication in intact taste buds through ATP signalling from pannexin 1 gap junction hemichannels. *J. Physiol.* 587 5899–5906 10.1113/jphysiol.2009.180083 19884319 PMC2808547

[B14] Dando R. Roper S. D. (2012). Acetylcholine is released from taste cells, enhancing taste signalling. *J. Physiol.* 590 3009–3017 10.1113/jphysiol.2012.232009 22570381 PMC3406387

[B15] Dotson C. D. Geraedts M. C. Munger S. D. (2013). Peptide regulators of peripheral taste function. *Semin. Cell Dev. Biol.* 24 232–239 10.1016/j.semcdb.2013.01.004 23348523 PMC3612364

[B16] Dvoryanchikov G. Huang Y. A. Barro-Soria R. Chaudhari N. Roper S. D. (2011). GABA, its receptors, and GABAergic inhibition in mouse taste buds. *J. Neurosci.* 31 5782–5791 10.1523/JNEUROSCI.5559-10.2011 21490220 PMC3320853

[B17] Finger T. E. (2005). Cell types and lineages in taste buds. *Chem. Senses *30(Suppl. 1) i54–i55 10.1093/chemse/bjh110 15738192

[B18] Finger T. E. Danilova V. Barrows J. Bartel D. L. Vigers A. J. Stone L. (2005). ATP signaling is crucial for communication from taste buds to gustatory nerves. *Science* 310 1495–1499 10.1126/science.1118435 16322458

[B19] Hall J. M. Hooper J. E. Finger T. E. (1999). Expression of sonic hedgehog, patched, and Gli1 in developing taste papillae of the mouse. *J. Comp. Neurol.* 406 143–155 10.1002/(SICI)1096-9861(19990405)406:2<143::AID-CNE1>3.0.CO;2-X 10096602

[B20] Hayato R. Ohtubo Y. Yoshii K. (2007). Functional expression of ionotropic purinergic receptors on mouse taste bud cells. *J. Physiol.* 584 473–488 10.1113/jphysiol.2007.138370 17702819 PMC2277161

[B21] Housley G. D. Bringmann A. Reichenbach A. (2009). Purinergic signaling in special senses. *Trends Neurosci.* 32 128–141 10.1016/j.tins.2009.01.001 19232752

[B22] Huang A. L. Chen X. Hoon M. A. Chandrashekar J. Guo W. Trankner D. (2006). The cells and logic for mammalian sour taste detection. *Nature* 442 934–938 10.1038/nature05084 16929298 PMC1571047

[B23] Huang Y. A. Dando R. Roper S. D. (2009). Autocrine and paracrine roles for ATP and serotonin in mouse taste buds. *J. Neurosci.* 29 13909–13918 10.1523/JNEUROSCI.2351-09.2009 19890001 PMC2821712

[B24] Huang Y. A. Grant J. Roper S. (2012). Glutamate may be an efferent transmitter that elicits inhibition in mouse taste buds. *PLoS ONE* 7:e30662 10.1371/journal.pone.0030662 PMC3266908 22292013

[B25] Huang Y. A. Maruyama Y. Roper S. D. (2008). Norepinephrine is coreleased with serotonin in mouse taste buds. *J. Neurosci.* 28 13088–13093 10.1523/JNEUROSCI.4187-08.2008 19052199 PMC3331792

[B26] Huang Y. A. Pereira E. Roper S. D. (2011a). Acid stimulation (sour taste) elicits GABA and serotonin release from mouse taste cells. *PLoS ONE* 6:e25471 10.1371/journal.pone.0025471 PMC3197584 22028776

[B27] Huang Y. A. Stone L. M. Pereira E. Yang R. Kinnamon J. C. Dvoryanchikov G. (2011b). Knocking out P2X receptors reduces transmitter secretion in taste buds. *J. Neurosci.* 31 13654–13661 10.1523/JNEUROSCI.3356-11.2011 21940456 PMC3188419

[B28] Huang Y. A. Roper S. D. (2010). Intracellular Ca^(2+)^ and TRPM5-mediated membrane depolarization produce ATP secretion from taste receptor cells. *J. Physiol.* 588 2343–2350 10.1113/jphysiol.2010.191106 20498227 PMC2915511

[B29] Huang Y. J. Maruyama Y. Dvoryanchikov G. Pereira E. Chaudhari N. Roper S. D. (2007). The role of pannexin 1 hemichannels in ATP release and cell–cell communication in mouse taste buds. *Proc. Natl. Acad. Sci. U.S.A.* 104 6436–6441 10.1073/pnas.0611280104 17389364 PMC1851090

[B30] Huang Y. J. Maruyama Y. Lu K. S. Pereira E. Plonsky I. Baur J. E. (2005). Mouse taste buds use serotonin as a neurotransmitter. *J. Neurosci.* 25 843–847 10.1523/JNEUROSCI.4446-04.2005 15673664 PMC6725637

[B31] Ishida Y. Ugawa S. Ueda T. Yamada T. Shibata Y. Hondoh A. (2009). P2X(2)- and P2X(3)-positive fibers in fungiform papillae originate from the chorda tympani but not the trigeminal nerve in rats and mice. *J. Comp. Neurol.* 514 131–144 10.1002/cne.22000 19266560

[B32] Iwatsuki K. Ichikawa R. Hiasa M. Moriyama Y. Torii K. Uneyama H. (2009). Identification of the vesicular nucleotide transporter (VNUT) in taste cells. *Biochem. Biophys. Res. Commun.* 388 1–5 10.1016/j.bbrc.2009.07.069 19619506

[B33] Iwatsuki K. Liu H. X. Gronder A. Singer M. A. Lane T. F. Grosschedl R. (2007). Wnt signaling interacts with Shh to regulate taste papilla development. *Proc. Natl. Acad. Sci. U.S.A.* 104 2253–2258 10.1073/pnas.0607399104 17284610 PMC1794217

[B34] Jia C. Cussen A. R. Hegg C. C. (2011). ATP differentially upregulates fibroblast growth factor 2 and transforming growth factor alpha in neonatal and adult mice: effect on neuroproliferation. *Neuroscience* 177 335–346 10.1016/j.neuroscience.2010.12.039 21187124 PMC3049987

[B35] Jung H. S. Oropeza V. Thesleff I. (1999). Shh, Bmp-2, Bmp-4 and Fgf-8 are associated with initiation and patterning of mouse tongue papillae. *Mech. Dev.* 81 179–182 10.1016/S0925-4773(98)00234-2 10330496

[B36] Kapsimali M. Barlow L. A. (2013). Developing a sense of taste. *Semin. Cell Dev. Biol.* 24 200–209 10.1016/j.semcdb.2012.11.002 23182899 PMC3604069

[B37] Kataoka S. Baquero A. Yang D. Shultz N. Vandenbeuch A. Ravid K. (2012). A2BR adenosine receptor odulates sweet taste in circumvallate taste buds. *PLoS ONE* 7:e30032 10.1371/journal.pone.0030032 PMC3254652 22253866

[B38] Kataoka S. Toyono T. Seta Y. Ogura T. Toyoshima K. (2004). Expression of P2Y(1) receptors in rat taste buds. *Histochem. Cell Biol.* 121 419–426 10.1007/s00418-004-0647-3 15103469

[B39] Kaya N. Shen T. Lu S. G. Zhao F. L. Herness S. (2004). A paracrine signaling role for serotonin in rat taste buds: expression and localization of serotonin receptor subtypes. *Am. J. Physiol. Regul. Integr. Comp. Physiol.* 286 R649–R658 10.1152/ajpregu.00572.2003 14715493

[B40] Lawton D. M. Furness D. N. Lindemann B. Hackney C. M. (2000). Localization of the glutamate-aspartate transporter, GLAST, in rat taste buds. *Eur. J. Neurosci.* 12 3163–3171 10.1046/j.1460-9568.2000.00207.x 10998100

[B41] Lazarowski E. R. Sesma J. I. Seminario L. Esther C. R. Jr. Kreda S. M. (2011). Nucleotide release by airway epithelia. *Subcell. Biochem.* 55 1–15 10.1007/978-94-007-1217-1_1 21560042

[B42] Liu F. Thirumangalathu S. Gallant N. M. Yang S. H. Stoick-Cooper C. L. Reddy S. T. (2007). Wnt-beta-catenin signaling initiates taste papilla development. *Nat. Genet.* 39 106–112 10.1038/ng1932 17128274

[B43] Mandadi S. Sokabe T. Shibasaki K. Katanosaka K. Mizuno A. Moqrich A. (2009). TRPV3 in keratinocytes transmits temperature information to sensory neurons via ATP. *Pflugers Arch.* 458 1093–1102 10.1007/s00424-009-0703-x 19669158 PMC2745623

[B44] Mistretta C. M. Liu H. X. Gaffield W. Maccallum D. K. (2003). Cyclopamine and jervine in embryonic rat tongue cultures demonstrate a role for Shh signaling in taste papilla development and patterning: fungiform papillae double in number and form in novel locations in dorsal lingual epithelium. *Dev. Biol.* 254 1–18 10.1016/S0012-1606(02)00014-3 12606278

[B45] Murata Y. Yasuo T. Yoshida R. Obata K. Yanagawa Y. Margolskee R. F. (2010). Action potential-enhanced ATP release from taste cells through hemichannels. *J. Neurophysiol.* 104 896–901 10.1152/jn.00414.2010 20519578 PMC3774670

[B46] Niki M. Takai S. Kusuhara Y. Ninomiya Y. Yoshida R. (2011). Responses to apical and basolateral application of glutamate in mouse fungiform taste cells with action potentials. *Cell Mol. Neurobiol.* 31 1033–1040 10.1007/s10571-011-9702-5 21573975 PMC11498453

[B47] Ohkuri T. Horio N. Stratford J. M. Finger T. E. Ninomiya Y. (2012). Residual chemoresponsiveness to acids in the superior laryngeal nerve in “taste-blind” (P2X2/P2X3 double-KO) mice. *Chem. Senses* 37 523–532 10.1093/chemse/bjs004 22362867 PMC3379842

[B48] Oka Y. Butnaru M. Von Buchholtz L. Ryba N. J. Zuker C. S. (2013). High salt recruits aversive taste pathways. *Nature* 494 472–475 10.1038/nature11905 23407495 PMC3587117

[B49] Piskuric N. A. Nurse C. A. (2013). Expanding role of ATP as a versatile messenger at carotid and aortic body chemoreceptors. *J. Physiol.* 591 415–422 10.1113/jphysiol.2012.234377 23165772 PMC3577521

[B50] Qiu F. Dahl G. (2009). A permeant regulating its permeation pore: inhibition of pannexin 1 channels by ATP. *Am. J. Physiol. Cell Physiol.* 296 C250–C255 10.1152/ajpcell.00433.2008 18945939 PMC2643853

[B51] Romanov R. A. Bystrova M. F. Rogachevskaya O. A. Sadovnikov V. B. Shestopalov V. I. Kolesnikov S. S. (2012). The ATP permeability of pannexin 1 channels in a heterologous system and in mammalian taste cells is dispensable. *J. Cell Sci.* 125 5514–5523 10.1242/jcs.111062 22956545 PMC3561859

[B52] Romanov R. A. Rogachevskaja O. A. Bystrova M. F. Jiang P. Margolskee R. F. Kolesnikov S. S. (2007). Afferent neurotransmission mediated by hemichannels in mammalian taste cells. *EMBO J.* 26 657–667 10.1038/sj.emboj.7601526 17235286 PMC1794384

[B53] Roper S. D. (2013). Taste buds as peripheral chemosensory processors. *Semin. Cell Dev. Biol.* 24 71–79 10.1016/j.semcdb.2012.12.002 23261954 PMC3690797

[B54] Royer S. M. Kinnamon J. C. (1988). Ultrastructure of mouse foliate taste buds: synaptic and nonsynaptic interactions between taste cells and nerve fibers. *J. Comp. Neurol. *270, 11–24 58–19 10.1002/cne.902700103 3372732

[B55] Shin Y. K. Martin B. Golden E. Dotson C. D. Maudsley S. Kim W. (2008). Modulation of taste sensitivity by GLP-1 signaling. *J. Neurochem.* 106 455–463 10.1111/j.1471-4159.2008.05397.x 18397368 PMC2629996

[B56] Starostik M. R. Rebello M. R. Cotter K. A. Kulik A. Medler K. F. (2010). Expression of GABAergic receptors in mouse taste receptor cells. *PLoS ONE* 5:e13639 10.1371/journal.pone.0013639 PMC2964312 21049022

[B57] Stone L. M. Finger T. E. (1994). Mosaic analysis of the embryonic origin of taste buds. *Chem. Senses* 19 725–735 10.1093/chemse/19.6.725 7735850

[B58] Taruno A. Vingtdeux V. Ohmoto M. Ma Z. Dvoryanchikov G. Li A. (2013). CALHM1 ion channel mediates purinergic neurotransmission of sweet, bitter and umami tastes. *Nature* 495 223–226 10.1038/nature11906 23467090 PMC3600154

[B59] Thorne P. R. Munoz D. J. Housley G. D. (2004). Purinergic modulation of cochlear partition resistance and its effect on the endocochlear potential in the guinea pig. *J. Assoc. Res. Otolaryngol.* 5 58–65 10.1007/s10162-003-4003-4 14976588 PMC2538371

[B60] Tomchik S. M. Berg S. Kim J. W. Chaudhari N. Roper S. D. (2007). Breadth of tuning and taste coding in mammalian taste buds. *J. Neurosci.* 27 10840–10848 10.1523/JNEUROSCI.1863-07.2007 17913917 PMC3717408

[B61] Vandenbeuch A. Anderson C. B. Ford A. P. Smith S. E. Finger T. E. Kinnamon S. C. (2013a). A selective P2X3, P2X2/3 receptor antagonist abolishes responses to all taste stimuli in mice. *Chem. Senses* 38 A645

[B62] Vandenbeuch A. Anderson C. B. Parnes J. Enjyoji K. Robson S. C. Finger T. E. (2013b). Role of the ectonucleotidase NTPDase2 in taste bud function. *Proc. Natl. Acad. Sci. U.S.A.* 110 14789–14794 10.1073/pnas.1309468110 23959882 PMC3767538

[B63] Vandenbeuch A. Clapp T. R. Kinnamon S. C. (2008). Amiloride-sensitive channels in type I fungiform taste cells in mouse. *BMC Neurosci.* 9:1 10.1186/1471-2202-9-1 PMC2235881 18171468

[B64] Vandenbeuch A. Tizzano M. Anderson C. B. Stone L. M. Goldberg D. Kinnamon S. C. (2010a). Evidence for a role of glutamate as an efferent transmitter in taste buds. *BMC Neurosci.* 11:77 10.1186/1471-2202-11-77 PMC2898831 20565975

[B65] Vandenbeuch A. Zorec R. Kinnamon S. C. (2010b). Capacitance measurements of regulated exocytosis in mouse taste cells. *J. Neurosci.* 30 14695–14701 10.1523/JNEUROSCI.1570-10.2010 21048127 PMC3064517

[B66] Yang R. Montoya A. Bond A. Walton J. Kinnamon J. C. (2012). Immunocytochemical analysis of P2X2 in rat circumvallate taste buds. *BMC Neurosci.* 13:51 10.1186/1471-2202-13-51 PMC3507709 22621423

[B67] Yarmolinsky D. A. Zuker C. S. Ryba N. J. (2009). Common sense about taste: from mammals to insects. *Cell* 139 234–244 10.1016/j.cell.2009.10.001 19837029 PMC3936514

[B68] Yee C. L. Yang R. Bottger B. Finger T. E. Kinnamon J. C. (2001). “Type III” cells of rat taste buds: immunohistochemical and ultrastructural studies of neuron-specific enolase, protein gene product 9.5, and serotonin. *J. Comp. Neurol.* 440 97–108 10.1002/cne.1372 11745610

[B69] Yoshida R. Miyauchi A. Yasuo T. Jyotaki M. Murata Y. Yasumatsu K. (2009). Discrimination of taste qualities among mouse fungiform taste bud cells. *J. Physiol.* 587 4425–4439 10.1113/jphysiol.2009.175075 19622604 PMC2766648

[B70] Zhang Y. Kolli T. Hivley R. Jaber L. Zhao F. I. Yan J. (2010). Characterization of the expression pattern of adrenergic receptors in rat taste buds. *Neuroscience* 169 1421–1437 10.1016/j.neuroscience.2010.05.021 20478367 PMC2914163

